# Sebaceous tumours: a prototypical class of skin tumour for universal germline genetic testing

**DOI:** 10.1111/bjd.20522

**Published:** 2021-07-22

**Authors:** R. Gallon, N. Kibbi, S. Cook, M. Santibanez‐Koref, M.S. Jackson, J. Burn, N. Rajan

**Affiliations:** ^1^ Translational and Clinical Research Institute Newcastle University Newcastle upon Tyne UK; ^2^ Department of Dermatology Stanford University Redwood City CA USA; ^3^ Department of Pathology Royal Victoria Infirmary Newcastle upon Tyne UK; ^4^ Biosciences Institute Newcastle University Newcastle upon Tyne UK; ^5^ Department of Dermatology Royal Victoria Infirmary Newcastle upon Tyne UK

dear editor, Lynch syndrome (LS) is an adult‐onset cancer predisposition caused by a germline pathogenic variant affecting one of four mismatch repair (MMR) genes (*MLH1*, *MSH2*, *MSH6* or *PMS2*). The vast majority of LS gene carriers are unknown, yet may benefit from risk‐reducing interventions such as targeted cancer screening and aspirin chemoprevention, the latter being associated with a persistent and long‐term reduction in colorectal cancer (CRC) incidence.^1^ Diagnostics guidance 27 from the UK National Institute for Health and Care Excellence recommends molecular analysis of all CRCs to screen for LS, with an expected yield of 3%. However, dermatologists treating patients with sebaceous tumours, including carcinomas and adenomas, may have a much higher chance of diagnosing LS, according to our recent review of LS screening strategies.^2^ In both settings, cascade testing family members will identify additional LS carriers. Here, we discuss this observation in more detail, as well as its implications for LS germline screening in patients with sebaceous carcinoma and benign sebaceous skin tumours.

Patients diagnosed with both sebaceous tumours and one or more LS‐related visceral malignancies are given a clinical diagnosis of Muir–Torre syndrome (MTS), a variant of LS most often caused by germline pathogenic variants in *MSH2*. However, sebaceous tumours may arise sporadically or occur in immunosuppressed organ transplant recipients (OTRs). The majority of sebaceous tumours in MTS are diagnosed subsequent to a visceral cancer, typically presenting in the sixth decade of life, and are clinically difficult to diagnose, warranting skin biopsy and histopathological examination. Sebaceous tumours are sentinel tumours in approximately 20% of MTS cases. LS‐related visceral malignancies are most commonly colorectal, endometrial, stomach and ovarian, although central nervous system, urothelial and small bowel cancers are also recognized. Like other LS tumours, MTS sebaceous tumours are characterized by MMR deficiency and increased microsatellite instability.^3^ Clinical practice guidelines from an expert panel for the management of sebaceous carcinoma recommend genetic testing for MTS in patients with extraocular sebaceous carcinoma and a Mayo MTS risk score ≥ 2 (Grade B recommendation), or patients aged < 50 years who do not meet the Mayo MTS risk score threshold but whose sebaceous carcinoma is MMR deficient (Grade D recommendation). Universal tumour immunohistochemistry (IHC) MMR deficiency testing of all sebaceous carcinomas was not recommended due to its much lower sensitivity and specificity for detecting LS compared with testing of CRCs.^4^

We collated estimates of LS incidence in different tumour types, using published studies that diagnosed LS by germline MMR gene testing without reported selection by clinical features, such as age or family history of cancer. Notably, the highest LS incidence was in patients with sebaceous tumours, at 33·3% (95% confidence interval 27·6–39·4) (Figure 1), with individual studies reporting incidences from 17·7% to 45·8%. The broad range between studies may be explained by distinct populations and study designs: the highest yield studies used patient cohorts from family cancer or genetics clinics; hence these patients represent a population in which the incidence of LS will be enriched. However, even the lowest estimated incidence of LS in patients with sebaceous tumours, at 17·7%, is still far greater than the estimated incidence in patients with CRC (Figure 1). In agreement with Owen *et␣al*.,^4^ we found a lower sensitivity and specificity (81·0% and 77·2%, respectively) of LS screening by tumour MMR deficiency testing in sebaceous tumours than in other Lynch‐spectrum tumour types.^2^

**Figure 1:**
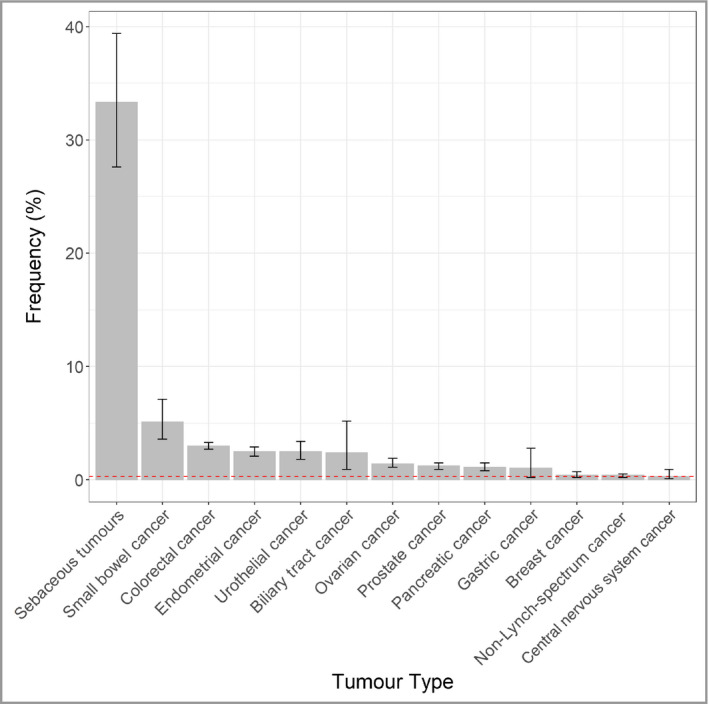
Frequency of Lynch syndrome (LS) among patients with different types of tumour. LS was diagnosed by detection of a germline pathogenic mismatch repair gene variant. Frequency estimates for each tumour type are based on patient populations without reported selection by clinical features, however, some studies used populations from family cancer or genetics clinics. The red dotted line represents an estimated frequency of LS in the general population of 0·3%.

Given the efficacy and affordability of aspirin as a cancer risk‐reducing intervention and the high frequency of LS diagnoses, there is a strong case for referral for LS investigation in patients with sebaceous tumours, particularly those with extraocular lesions. The poor sensitivity and specificity of screening by MMR IHC of sebaceous tumours, and the exceptional LS incidence in this population, favours reflex germline MMR gene testing, although parallel molecular analysis of tumours may be pertinent for variant interpretation. Indeed, germline *BRCA* gene testing is routinely offered to all UK patients with ovarian cancer, due to germline pathogenic variants in *BRCA1*/*BRCA2* being found in approximately 15% of patients and the interventions that can be offered.^5^ A request for germline testing to be initiated immediately, with referral for genetic counselling if a pathogenic variant is reported, was shown to be well received by patients. Therefore, we reason that all patients, other than OTRs, diagnosed with extraocular sebaceous tumours should be offered germline genetic testing to identify LS, with clinical benefit for patients and their relatives.

## Author Contribution

**Richard Gallon:** Conceptualization (equal); Data curation (equal); Investigation (equal); Methodology (equal); Visualization (equal); Writing‐original draft (equal); Writing‐review & editing (equal). **Nour Kibbi:** Conceptualization (equal); Data curation (equal); Methodology (equal); Writing‐original draft (equal); Writing‐review & editing (equal). **Sam Cook:** Conceptualization (equal); Data curation (equal); Validation (equal); Writing‐original draft (equal); Writing‐review & editing (equal). **Mauro Santibanez‐Koref:** Conceptualization (equal); Supervision (equal); Writing‐original draft (equal); Writing‐review & editing (equal). **Michael Jackson:** Conceptualization (equal); Supervision (equal); Writing‐original draft (equal); Writing‐review & editing (equal). **John Burn:** Conceptualization (equal); Supervision (equal); Writing‐original draft (equal); Writing‐review & editing (equal). **Neil N Rajan:** Conceptualization (equal); Project administration (equal); Supervision (equal); Writing‐original draft (equal); Writing‐review & editing (equal).
